# Experimental Validation of a Distributed 5G Core with Store-and-Forward for IoT Sensing over LEO Non-Terrestrial Networks

**DOI:** 10.3390/s26154919

**Published:** 2026-08-04

**Authors:** Victor Monzon Baeza, Francesc Xavier Romero Soto, Raúl Parada, Carlos Monzo

**Affiliations:** 1 Faculty of Computer Science, Multimedia and Telecommunications, Universitat Oberta de Catalunya, Rambla del Poblenou 156, 08018 Barcelona, Spaincmonzo@uoc.edu (C.M.); 2 Centre Tecnològic de Telecomunicacions de Catalunya, Av. Carl Friedrich Gauss, 7-Edifici B4, 08860 Castelldefels, Spain; rparada@cttc.es

**Keywords:** non-terrestrial networks, LEO satellites, distributed 5G core, store-and-forward, delay-tolerant IoT, system architecture, experimental validation

## Abstract

Low Earth Orbit (LEO) Non-Terrestrial Networks (NTNs) are emerging as a promising connectivity solution for Internet of Things (IoT) sensing applications deployed in remote, isolated, or infrastructure-limited environments. However, sparse LEO constellations inherently lead to intermittent connectivity, long service gaps, and frequent disruptions, challenging conventional 5G architectures that assume continuous end-to-end availability. This paper presents and experimentally validates a distributed 5G Core architecture enhanced with Store-and-Forward (S&F) capabilities to enable reliable delivery of IoT sensing data over intermittently connected LEO-NTN scenarios. The proposed architecture distributes selected 5G Core functions between ground and satellite nodes and introduces an S&F module capable of locally buffering uplink IoT data during periods without feeder-link connectivity and forwarding them once the ground connection is restored. A functional prototype is implemented using Open5GS, UERANSIM, and an emulated satellite node, and experimentally evaluated under representative intermittent-connectivity conditions. The results demonstrate that the proposed architecture successfully preserves and delivers IoT sensing data across temporary link disruptions. The experimental findings confirm the feasibility of integrating S&F mechanisms into distributed 5G Core architectures and provide practical insights into the design of resilient IoT sensing services over sparse LEO constellations.

## 1. Introduction

The extension of mobile connectivity beyond terrestrial infrastructures has become a key enabler for large-scale Internet of Things (IoT) deployments in remote, rural, and infrastructure-less environments. In this context, Non-Terrestrial Networks (NTNs) based on Low Earth Orbit (LEO) satellite constellations have emerged as a viable solution to provide global coverage and support services in scenarios where continuous end-to-end connectivity cannot be guaranteed [1]. However, the dynamic topology, intermittent visibility, and frequent backhaul disruptions inherent to LEO systems fundamentally challenge the centralized assumptions of conventional mobile network architectures.

In parallel, Delay-Tolerant Networking (DTN) and Store-and-Forward (S&F) mechanisms have gained renewed attention as a practical approach to cope with intermittent connectivity, long propagation delays, and disrupted backhaul conditions. Such characteristics are increasingly relevant not only in satellite systems, but also in rural connectivity, disaster recovery, and mobile sensing scenarios [2,3]. S&F mechanisms enable reliable service operation even in the absence of continuous connectivity, making them a cornerstone for future mobile and satellite-integrated networks.

Standard fifth-generation (5G) core network designs rely on persistent connectivity between the Radio Access Network (RAN) and centralized core functions to ensure continuous control-plane coordination and user-plane data delivery. In LEO-based NTN scenarios, this assumption is frequently violated due to satellite mobility and limited contact windows, particularly in sparse constellations and IoT-oriented deployments [4,5]. As a result, supporting reliable IoT services in NTN requires architectural adaptations that enable autonomous operation during connectivity outages while preserving end-to-end service continuity and compliance with 3GPP specifications. Several recent studies have explored S&F operation in satellite-based IoT systems from different perspectives. Architectural proposals for S&F-enabled NTN highlight the role of onboard buffering and intermediate user-plane functions to tolerate discontinuous feeder links [6]. Other works focus on mitigating latency and improving delivery performance through techniques such as inter-satellite links (ISLs), further reinforcing the relevance of S&F in LEO constellations [7]. Experimental evaluations of delay-tolerant communication have been conducted in terrestrial and hybrid environments, demonstrating the feasibility and robustness of DTN-based approaches under real connectivity constraints [2].

However, the majority of system-level experimental studies on satellite IoT rely on Low-Power Wide-Area Network (LPWAN) technologies, particularly Long Range (LoRa) or LoRaWAN. Multiple works analyze store-and-forward performance, buffering behavior, and delivery ratios in LoRaWAN-based satellite systems, highlighting their suitability for non-real-time IoT services with extremely low data rates [8,9]. While these approaches effectively demonstrate the feasibility of S&F over satellite links, they remain largely detached from the evolution of standardized mobile core networks and offer limited integration with 3GPP-compliant architectures.

In contrast, recent standardization efforts within 3GPP have explicitly addressed the integration of satellite components into the 5G system. In particular, Technical Report TR 23.700-29 (Release 19) [4] identifies support for delay-tolerant services as a key issue for NTN-enabled IoT deployments and outlines architectural principles for distributing selected core network functions across satellite and terrestrial segments. Nevertheless, these specifications primarily provide high-level architectural guidance, leaving open questions regarding practical system design, functional placement, and experimental validation.

In this context, this paper addresses the following research question: *to what extent can a distributed 5G core architecture with integrated Store-and-Forward capabilities support autonomous IoT operation over LEO-based NTN while remaining compliant with existing 3GPP frameworks?* Rather than focusing on performance optimization, the objective of this work is to assess the system-level feasibility, architectural dependencies, and design trade-offs associated with distributing 5G core network functions in intermittently connected environments.

To this end, the paper presents a distributed 5G core system architecture in which selected control-plane and user-plane functions are deployed across satellite and terrestrial segments, enabling localized autonomy and in-space data persistence during backhaul disruptions. A representative system prototype is used to experimentally validate end-to-end operation across disconnected operational phases, allowing the analysis of functional interactions, operational states, and architectural constraints under realistic NTN conditions.

The main contributions of this paper are summarized as follows:

- We present a system-level design of a distributed 5G core architecture for IoT services over LEO-based NTN, addressing intermittent connectivity through integrated S&F mechanisms.
- We analyze the functional distribution of 5G core network functions between satellite and terrestrial segments, identifying architectural dependencies and design trade-offs required to enable autonomous operation during backhaul disruptions.
- We experimentally validate the proposed system using a representative prototype, demonstrating autonomous registration, session establishment, and reliable store-and-forward data delivery across disconnected operational phases.
- We derive practical design insights aligned with 3GPP Release 19 NTN principles, highlighting both the feasibility and limitations of deploying resilient, delay-tolerant IoT services using distributed 5G core architectures.

The remainder of this paper is organized as follows. Section 2 shows the related works. Section 3 discusses the system requirements and design challenges for distributed 5G core deployment in NTN scenarios. Section 4 presents the proposed system architecture, while Section 5 describes the implementation of the representative system prototype. Section 6 analyzes the experimental results from a system-level perspective. Finally, Section 7 concludes the paper and outlines directions for future work.

## 2. Related Work

S&F mechanisms have long been studied as a fundamental approach to support delay-tolerant communications in satellite networks. Early research focused primarily on routing strategies, buffering policies, and link-level considerations in LEO constellations. For instance, Lu et al. [10] analyzed the routing complexity of S&F-enabled LEO satellite networks, highlighting the impact of satellite mobility on data delivery paths and forwarding delays. Similarly, Rahim et al. [11] investigated link budget and system design aspects for ground sensor terminals in nanosatellite-based S&F missions. While these works effectively address S&F operations in satellite systems, they do not consider their integration into 5G core network functions; in contrast, this paper explicitly integrates S&F mechanisms into a distributed 5G core architecture and validates their operation at the system level.

Standardization efforts within the 3rd Generation Partnership Project (3GPP) have explicitly recognized these challenges. In particular, TR 23.700-29 (Release 19) identifies the support of delay-tolerant services as a key issue for NTN-enabled IoT deployments and outlines high-level architectural principles for distributing network functions across satellite and terrestrial segments. Nevertheless, the specification does not provide guidance on practical implementation strategies or experimental validation, leaving open questions regarding system feasibility and operational behavior.

Kellermann et al. propose a high-level S&F-enabled NTN architecture and report a laboratory validation for narrowband-IoT (NB-IoT)/Evolved Packet Core (EPC)-oriented components, focusing on feasibility and standard-compliant operation under discontinuous feeder links [12]. In that architecture, the core network is still based on 4G. In contrast, our paper takes a *system design and implementation* perspective to provide a complete end-to-end prototype of a split 5G core across satellite and terrestrial segments and to characterize its operational behavior under intermittent backhaul, including autonomous operation, data persistence, and deferred forwarding.

From a standardization/procedure perspective, [13] analyzes how S&F can be supported in multi-satellite deployments by identifying impacted entities and proposing adaptations to core procedures (e.g., registration continuation across contacts and S&F-aware data transfer). That work is intentionally oriented toward the impact of procedures and the behavior of the conceptual system. The contribution of the present paper is complementary and goes one step further: we demonstrate a *working system* that operationalizes these concepts into an implementable split-core design, and we validate it experimentally at the system level, emphasizing repeatable end-to-end functionality rather than protocol proposal alone.

Other related works address narrower functional slices of the S&F NTN problem. Zong et al. discuss architecture options and propose enhancements to mobility/session management procedures (e.g., S&F mode broadcasting, attach/TAU/PDN connectivity) for split-MME and full-EPC onboard deployments [9]. Saglam proposes an enhanced paging method for S&F IoT-NTN and evaluates it via simulation, focusing on paging triggering and energy/signaling aspects at the access layer [14]. On the other hand, our work concentrates on *system-level integration and validation* of a split-core design with store-and-forward service logic, providing an operational model (state transition) and experimental evidence that the system behaves correctly across disconnection and reconnection phases.

Overall, the novelty of this paper is not a new paging rule or a set of message/procedure updates, but a *systems-oriented* contribution: (i) an end-to-end split-core S&F design suitable for intermittent NTN backhaul, (ii) an operational model that captures autonomous and synchronized modes, and (iii) a system-level experimental validation that demonstrates autonomous operation, data persistence, connectivity restoration, and end-to-end delivery under realistic disconnection assumptions.

## 3. System Requirements and Design Challenges

Supporting IoT services over LEO satellite-based NTN introduces a set of system requirements that differ significantly from those assumed in conventional terrestrial 5G deployments. The combination of intermittent connectivity, dynamic network topology, and limited satellite resources requires architectural adaptations at the core network level to ensure reliable and resilient service delivery. This section outlines the key system requirements and design challenges that motivate the proposed distributed 5G core architecture with integrated S&F capabilities.

### 3.1. Intermittent Connectivity and Autonomous Operation

LEO satellite constellations inherently provide time-varying coverage due to satellite mobility and constrained visibility windows. As a consequence, continuous connectivity between the Radio Access Network (RAN) and centralized core network functions cannot be guaranteed, particularly in sparse constellations and IoT-oriented scenarios. From a system perspective, this intermittent connectivity imposes the requirement for autonomous operation during backhaul disruptions.

To maintain service continuity under these conditions, selected control-plane functions must be able to operate independently of the terrestrial core for extended periods. This includes supporting User Equipment (UE) registration, mobility management, and session establishment without relying on persistent connectivity to centralized network functions. Enabling such autonomy challenges the traditional centralized 5G core deployment model and motivates distributing core network functions across satellite and terrestrial segments.

### 3.2. Delay-Tolerant Data Delivery and S&F Support

A wide range of IoT applications targeted by NTN deployments, such as environmental monitoring, asset tracking, and remote sensing, are inherently delay-tolerant. However, while these applications can tolerate delivery delays, they typically require reliable data persistence to prevent information loss during connectivity outages.

This requirement introduces the need for Store-and-Forward mechanisms capable of decoupling data generation from data delivery. From a system design standpoint, S&F functionality must be tightly integrated with the core network architecture to ensure compatibility with 5G procedures and to enable seamless data forwarding once connectivity is restored. Designing such mechanisms raises challenges related to data persistence, message integrity, and coordination between autonomous and centralized network segments.

### 3.3. Functional Distribution and Architectural Dependencies

Distributing 5G core network functions across satellite and terrestrial segments requires careful consideration of functional dependencies and signaling interactions. While some functions can tolerate intermittent connectivity and operate autonomously, others rely on global network state, centralized policy enforcement, or continuous synchronization.

From a system-level perspective, determining which functions can be deployed in the satellite segment without compromising network integrity is a key design challenge. This includes analyzing dependencies between access and mobility management, session management, user-plane handling, and service discovery. Improper functional placement may lead to inconsistencies, increased signaling overhead, or service disruption upon reconnection. Therefore, the functional distribution must balance autonomy, complexity, and compliance with existing 5G architectural principles.

### 3.4. Resource Constraints in the Satellite Segment

Satellite platforms impose strict constraints on computational resources, storage capacity, and energy consumption. Unlike terrestrial data centers, satellite-based deployments must operate within limited and often fixed resource budgets. These constraints directly influence the feasibility of deploying core network functions in space.

From a system design perspective, this limitation motivates, at the architectural level, selective core-function deployment rather than full core replication: only a reduced control set needs to be active on board during autonomous operation. In the prototype this reduced set is instantiated as part of a full Open5GS deployment for tooling simplicity, as clarified in Section 5.5. The architecture must ensure that the satellite segment can sustain autonomous operation during disconnections without exceeding resource limitations, while still enabling seamless reintegration with the terrestrial core once connectivity is restored.

### 3.5. Compliance with 3GPP Architectural Principles

Any architectural adaptation for NTN deployments must preserve compatibility with 3GPP specifications to ensure interoperability and future extensibility. Recent 3GPP studies, particularly TR 23.700-29 (Release 19) [4], recognize the need for distributed architectures and delay-tolerant support in NTN scenarios but leave open questions regarding practical implementation and system behavior.

A key challenge is therefore to translate these high-level architectural principles into a concrete system design that remains compliant with standard 5G procedures. This includes maintaining standard registration and session management workflows, ensuring proper service discovery across distributed components, and avoiding proprietary protocol extensions. Addressing these challenges is essential to assessing the practical feasibility of distributed 5G core architectures for NTN-enabled IoT services.

## 4. Distributed 5G Core System Architecture

This section presents the proposed distributed 5G core system architecture designed to support delay-tolerant IoT services over LEO NTN. The architecture directly addresses the system requirements and design challenges identified in Section 3 by enabling autonomous operation during intermittent connectivity, integrating S&F mechanisms, and preserving compliance with 3GPP architectural principles.

### 4.1. Architecture Overview

**Figure 1:**
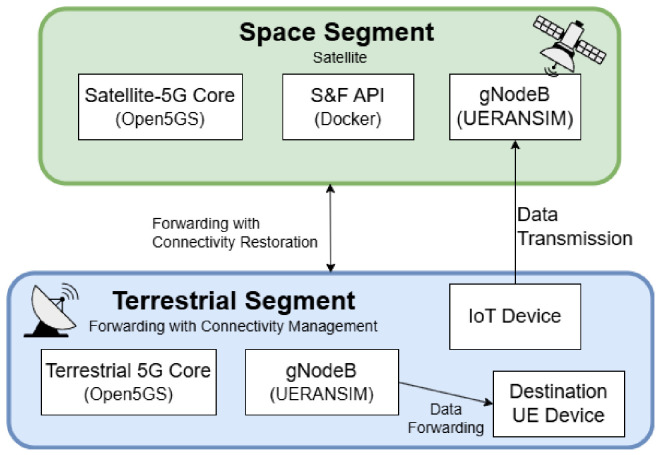
Split 5G core architecture with S&F mechanisms for IoT-NTN communications.

Figure 1 illustrates the proposed split 5G core architecture in which selected core network functions are distributed between satellite and terrestrial segments. Rather than replicating the full 5G core in space, the architecture requires only a reduced set of essential control-plane and user-plane functions to be active in the satellite segment during autonomous operation, while maintaining a complete core network in the terrestrial segment. In the prototype, this reduced set is instantiated within a full Open5GS deployment for tooling simplicity, and only the reduced subset is functionally exercised on the delay-tolerant path (see Section 5.5). This division is deliberately designed to balance autonomy, complexity, and resource efficiency on three grounds: (i) the functions kept in the satellite segment (AMF, SMF, UPF and the S&F service) are exactly those needed to run registration, authentication and PDU-session establishment during a feeder-link outage, i.e., the minimal set that preserves autonomy; (ii) complexity stays low because the satellite AMF federates with the terrestrial core through the standard service-based architecture and NRF discovery, without introducing new interfaces, unlike earlier EPC-based store-and-forward proposals; and (iii) resource use is contained because centralized coordination, policy control and service exposure remain on the ground. The split is therefore the deliberate middle point between two extremes it avoids: full core replication in space (maximum autonomy, maximum cost) and a transparent bent-pipe relay (no cost, no autonomy).

The satellite segment is designed to operate independently during backhaul disruptions, supporting user UE access, session establishment, and local data handling. The terrestrial segment provides centralized coordination, service exposure, and final data delivery once connectivity is restored. Inter-segment communication follows standard 5G service-based interfaces, enabling seamless reintegration without modifying existing procedures.

### 4.2. Satellite Segment Architecture

The satellite segment hosts the network functions required to sustain autonomous operation under intermittent connectivity conditions. In particular, access and mobility management and session control capabilities are locally available to support UE registration and session establishment without reliance on the terrestrial core.

To address delay-tolerant data delivery requirements, a Store-and-Forward service is integrated within the satellite segment. This service provides persistent storage for IoT data generated during connectivity outages and manages controlled forwarding once backhaul connectivity becomes available. By collocating data persistence with control-plane autonomy, the satellite segment can ensure service continuity while decoupling data generation from end-to-end delivery.

The satellite segment is designed as a modular subsystem, allowing independent operation and recovery. This modularity simplifies fault isolation and limits the impact of disconnections on the overall system, while enabling flexible scaling based on mission requirements and resource availability.

To manage forwarding on link-up, the S&F service also includes a monitoring component designed to detect restored feeder-link connectivity and forward the buffered messages toward the terrestrial segment. As discussed in Section 6, the autonomous operation of this component was not captured in the reported experiments; the delivery path evidenced here is the retrieval of the persisted messages by the destination.

### 4.3. Terrestrial Segment Architecture

The terrestrial segment hosts the full 5G core network and is responsible for global coordination, policy enforcement, and service exposure. It maintains authoritative network state and provides access to external data networks and application services.

Upon restoration of connectivity, the terrestrial core interacts with the satellite segment to synchronize network functions and receive stored IoT data. Service discovery mechanisms enable dynamic inter-segment interaction, allowing distributed functions to register, expose, and consume services using standard interfaces. This design ensures that the distributed architecture remains transparent to end applications and does not require modifications to existing service workflows.

### 4.4. Inter-Segment Interaction and Service Continuity

Inter-segment interaction is a key aspect of the proposed architecture. During connectivity outages, the satellite segment operates autonomously, maintaining local network state and data persistence. Once backhaul connectivity is restored, the system transitions to a synchronized operational mode in which stored data is forwarded and network functions reestablish coordination with the terrestrial core.

This transition is designed to be seamless and transparent to user equipment. Standard 5G procedures are preserved throughout the process, avoiding the need for proprietary signaling extensions. The architecture, therefore, enables continuous service support across disconnected operational phases while maintaining compliance with 3GPP-defined workflows.

### 4.5. Architectural Alignment with System Requirements

The proposed distributed architecture (Figure 1) directly addresses the system requirements identified in Section 3. Autonomous operation is enabled through local control-plane deployment in the satellite segment, while delay-tolerant data delivery is achieved via integrated Store-and-Forward mechanisms. Functional distribution decisions are guided by architectural dependencies and resource constraints, so that only the control subset required for autonomous operation needs to be active in the satellite segment rather than a replicated centralized core. Finally, the use of standard service-based interfaces ensures compatibility with 3GPP architectural principles and supports future extensibility.

## 5. System Prototype and Implementation

To assess the practical feasibility of the proposed distributed 5G core architecture, a representative system prototype was implemented and deployed in a controlled experimental environment. The objective of this prototype is not performance optimization, but rather the validation of system-level behavior, functional interactions, and architectural assumptions under intermittent connectivity conditions representative of LEO-based NTN.

The prototype implements the distributed architecture described in Section 3 by instantiating separate satellite and terrestrial segments interconnected through standard 5G interfaces. Each segment hosts a distinct set of core network functions consistent with the proposed functional distribution, enabling autonomous operation during connectivity disruptions and coordinated operation once backhaul connectivity is restored.

The representative system prototype was implemented using open-source 5G platforms to support architectural validation. Core network functions were realized using Open5GS [15], while UE and gNodeB behavior was emulated using UERANSIM [16]. The system is deployed using virtualized computing environments to emulate the separation between satellite and terrestrial domains. This approach enables controlled experimentation while preserving architectural fidelity and reproducibility, ensuring alignment with 3GPP architectural principles.

### 5.1. Satellite Segment Implementation

The satellite segment of the prototype is designed to support autonomous operation during periods of terrestrial disconnection. It hosts a 5G core configuration that provides access and mobility management, session control, and local user-plane handling for IoT devices. This configuration enables UE registration, authentication, and Protocol Data Unit Session (PDU) session establishment without reliance on continuous backhaul connectivity.

To support delay-tolerant data delivery, an S&F service is integrated within the satellite segment. The S&F service provides persistent storage for IoT data generated during connectivity outages and exposes controlled interfaces for data insertion, retrieval, and forwarding. Data persistence ensures that information generated by IoT devices is preserved until terrestrial connectivity becomes available, decoupling data generation from end-to-end delivery.

The satellite segment is deployed as a modular subsystem, allowing independent initialization, operation, and recovery. This modularity reflects the design objective of limiting interdependencies during disconnected operation and reducing the impact of backhaul disruptions on local service availability.

### 5.2. Terrestrial Segment Implementation

The terrestrial segment hosts a complete 5G core network configuration and represents the centralized coordination domain of the system. It maintains authoritative network state, supports service discovery, and provides connectivity to external data networks and application services.

Upon restoration of backhaul connectivity, the terrestrial core interacts with the satellite segment to reestablish coordination and receive stored IoT data. Standard service-based interfaces enable distributed network functions to register, discover services, and exchange control-plane information without requiring manual intervention or proprietary extensions. This design ensures that the transition from autonomous to synchronized operation remains transparent to user equipment and external services.

### 5.3. Inter-Segment Connectivity and Operational Phases

**Figure 2:**
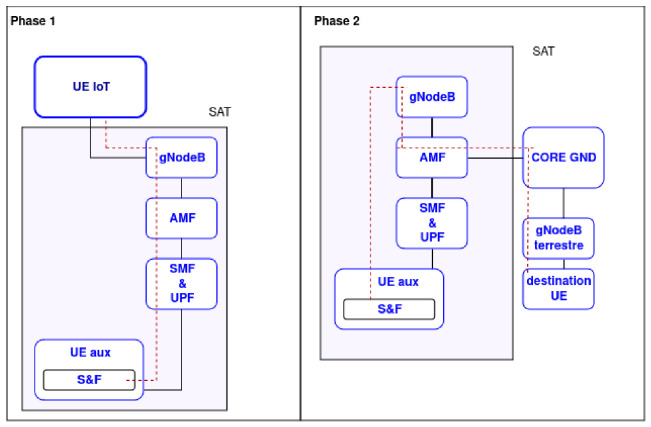
Operational phases of the distributed 5G core system.

The prototype explicitly supports two operational phases, as illustrated in Figure 2, reflecting the intermittent connectivity characteristics of LEO-based NTN deployments and the corresponding system behavior. The figure abstracts the distributed core architecture into two representative modes of operation and highlights the role of local core functions and auxiliary entities during each phase.

In the first phase, the satellite segment operates autonomously, without any assumed connectivity to the terrestrial core. During this phase, essential control-plane functions, including Access and Mobility Management Function (AMF) and Session Management Function (SMF), are locally available within the satellite segment to support UE registration, authentication, and PDU session establishment. IoT devices access the network through the satellite gNodeB, and user-plane traffic is handled locally. The auxiliary UE depicted in Figure 2 serves as an intermediary to emulate S&F interaction and local data persistence within the satellite segment, enabling controlled validation of disconnected operation.

In the second phase, backhaul connectivity between satellite and terrestrial segments is restored. The system transitions to a synchronized operational mode in which control-plane coordination is reestablished through standard 5G service discovery mechanisms. Deferred data stored during autonomous operation is forwarded toward the terrestrial core and subsequently delivered to external services or destination UEs. Throughout this transition, standard 5G workflows are preserved, and no protocol extensions or proprietary signaling mechanisms are introduced.

By explicitly supporting these two operational phases, the prototype demonstrates that intermittent connectivity can be accommodated through architectural design rather than protocol modification. Figure 2, therefore, serves as a conceptual reference for the operational behavior validated experimentally in the subsequent sections.

### 5.4. Reproducibility and Experimental Scope

The prototype is implemented using open-source 5G platforms and containerized services to facilitate reproducibility and transparency. Virtualized deployment enables precise control over network topology, functional placement, and connectivity conditions. While the prototype does not aim to replicate the physical constraints of an actual satellite platform, it provides a faithful representation of the functional and architectural behavior required to assess system-level feasibility.

The scope of the experimental setup is intentionally limited to validating architectural operation, functional interaction, and service continuity across disconnected phases. Performance optimization, large-scale scalability, and physical-layer effects are outside the scope of this study and are identified as directions for future work.

### 5.5. Testbed Configuration and Reproducibility

The distributed prototype is deployed over five virtual machines interconnected through an isolated management network. Table 1 details the hardware and software of the testbed, and Table 2 lists the role and management address of each virtual machine. Each 5G core segment runs an independent set of Open5GS network functions with the service-based interface (SBI) configuration reported in Table 3, while access nodes and terminals are emulated with UERANSIM. Three subscribers (Table 4) reproduce the end-to-end path: an originating IoT UE in the satellite segment, an auxiliary UE that mediates local Store-and-Forward persistence, and a destination UE in the terrestrial segment. The complete mapping between roles, management addresses and dynamically assigned PDU-session addresses is given in Table 5, and the REST interface exposed by the S&F service is summarized in Table 6. Intermittent connectivity is emulated in a controlled and repeatable manner by starting and stopping the corresponding services and virtual machines, reproducing the loss and recovery of the feeder link without requiring a physical channel or orbital model.

Although Table 3 lists the full Open5GS network-function set in both segments, this reflects a deployment choice: the complete set is instantiated per segment for tooling simplicity and reproducibility, while only the control subset required for autonomous operation (AMF, SMF and UPF) together with the S&F service is exercised on the delay-tolerant path in the satellite segment. Open5GS allows starting only the required functions, so a minimized onboard deployment is directly supported and does not alter the proposed architecture.

## 6. Experimental Results and System-Level Analysis

This section presents the experimental results obtained using the system prototype described in Section 5. The objective of the analysis is to assess the system-level behavior of the proposed distributed 5G core architecture under intermittent connectivity conditions, rather than to optimize performance or conduct large-scale benchmarking. All results are derived from the experimental setup described in [13] and focus on validating architectural feasibility, functional interaction, and service continuity.

### 6.1. Experimental Configuration and Validation Scope

The experimental setup is intentionally designed to reflect a realistic separation between satellite and terrestrial segments, enabling the evaluation of autonomous operation, local data persistence, and deferred data forwarding. Table 7 summarizes the key configuration aspects of the experimental system, highlighting the functional roles of each network segment, the operational modes considered, and the scope of data persistence evaluated during the experiments. Rather than providing exhaustive implementation details, the table contextualizes the validation environment and clarifies the assumptions under which system-level behavior is assessed.

Detailed reproducibility parameters of this environment, testbed hardware and software, virtual-machine roles, network-function SBI configuration, subscriber identities, and Store-and-Forward endpoints, are reported in Section 5.5 (Table 1, Table 2, Table 3, Table 4, Table 5 and Table 6).

The validation process focuses on two operational modes: an autonomous satellite operation mode, in which the satellite segment operates without any assumed backhaul connectivity, and a synchronized operation mode, in which coordination with the terrestrial core is restored. These modes form the basis for all experimental scenarios discussed in the following subsections and are representative of typical connectivity conditions encountered in LEO satellite deployments.

**Figure 3:**
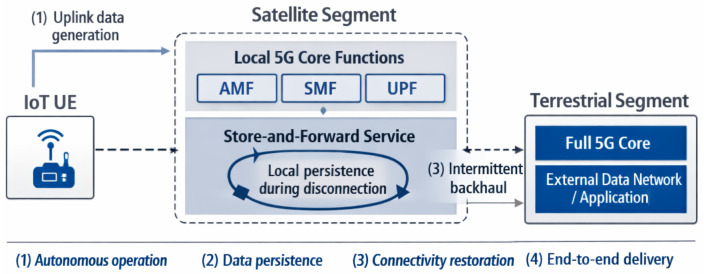
Functional validation flow of the distributed 5G core system over LEO-based NTN. The satellite segment operates autonomously to support UE access and locally persists IoT data via the S&F service during backhaul outages. When connectivity is restored, deferred data is forwarded to the terrestrial segment for end-to-end delivery.

Figure 3 provides a consolidated view of the end-to-end functional validation flow used throughout this study and serves as a reference framework for interpreting the experimental results. The figure illustrates how IoT data generation, local core operation, S&F persistence, and deferred delivery are coordinated across satellite and terrestrial segments.

During autonomous operation, essential 5G core functions, including AMF, SMF, and user-plane handling (UPF), are locally available within the satellite segment to support UE access, registration, and PDU session establishment. User-plane data generated by IoT devices is handled locally and persisted via the S&F service, enabling reliable operation despite the absence of backhaul connectivity. This behavior is validated experimentally and analyzed in Section 6.2 and Section 6.3.

Upon restoration of backhaul connectivity, the system transitions to a synchronized operational mode. Deferred data stored during autonomous operation is forwarded toward the terrestrial segment, and control-plane coordination with the centralized core is reestablished using standard 5G service discovery mechanisms. This phase enables end-to-end data delivery to external services or destination UEs without requiring protocol extensions or proprietary signaling. The reintegration process and its system-level implications are examined in Section 6.4 and Section 6.5.

By structuring the experimental analysis around the functional blocks illustrated in Figure 3 and the configuration summarized in Table 7, the results demonstrate that the proposed architecture exhibits consistent, repeatable, and standards-compliant behavior across disconnected and reconnected operational phases.

### 6.2. Autonomous Operation of the Satellite Segment

The first set of experiments validates the ability of the satellite segment to operate autonomously during periods of terrestrial disconnection. When backhaul connectivity is unavailable, the satellite segment initializes its local core network functions and provides access services to IoT UE without relying on external coordination.

Experimental observations confirm that essential control-plane functions remain operational throughout the disconnected phase, enabling uninterrupted local service support. This behavior demonstrates that the architectural distribution of core functions effectively satisfies the autonomy requirement identified in Section 3.1. and confirms the feasibility of isolated operation in LEO-based NTN scenarios.

From an experimental perspective, UE registration and PDU-session establishment procedures completed in approximately 0.35 s (a registration procedure of about 70 ms followed by a PDU-session establishment of about 280 ms), as measured from the log timestamps in Figure 6. These values are not intended as performance benchmarks, but rather as representative indicators confirming that standard 5G control-plane procedures can be executed reliably in the satellite segment despite the absence of continuous backhaul connectivity.

**Figure 4:**
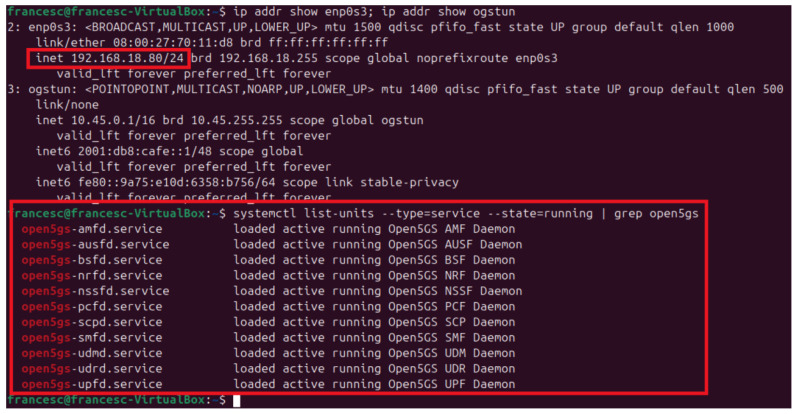
Satellite core: configured IP and active NF services.

**Figure 5:**
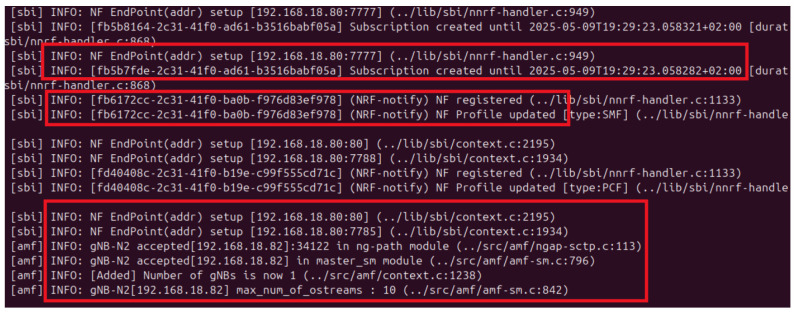
Successful satellite gNodeB registration at the AMF.

Across all repetitions, the satellite core (Figure 4) initialized every network function and accepted the registration of the satellite gNodeB (Figure 5) over the SCTP/NG interface, confirming that autonomous bring-up of the local core is achieved without any terrestrial coordination.

### 6.3. UE Registration and Session Establishment

To further validate control-plane continuity, UE registration and PDU session establishment procedures were evaluated during autonomous operation. IoT UEs successfully completed standard 5G registration workflows, including authentication, security context establishment, and session creation.

The experimental results show that registration procedures remain consistent with standard 5G behavior, with UEs transitioning through expected mobility management states and receiving valid session parameters. These observations confirm that distributing selected control-plane functions to the satellite segment does not disrupt standard registration or session management processes, supporting compliance with 3GPP-defined workflows.

**Figure 6:**
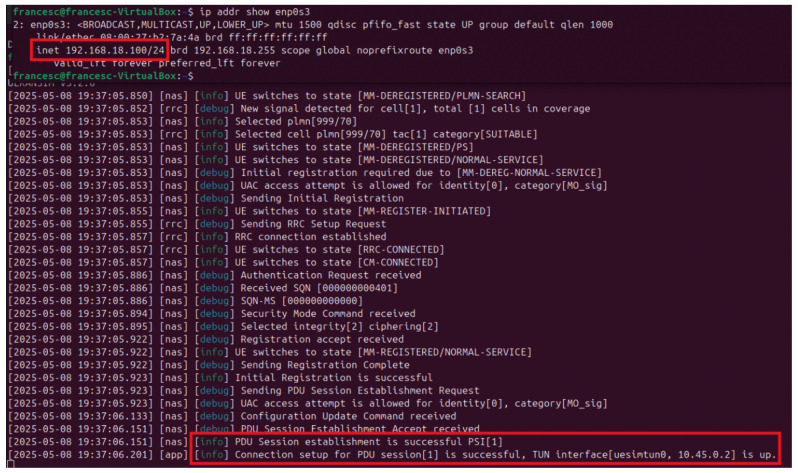
IoT UE registration and PDU session (IMSI and session IP).

**Figure 7:**
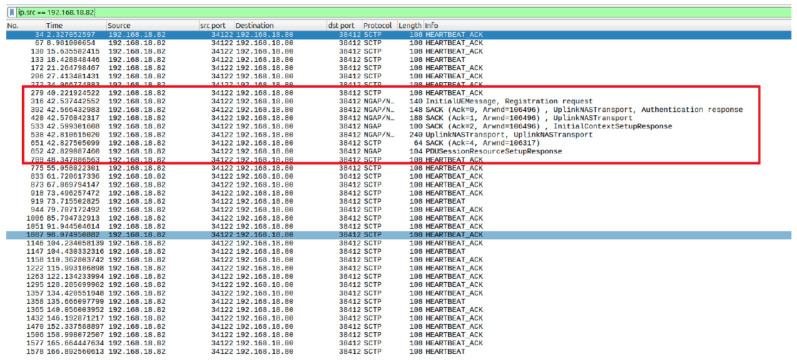
Wireshark capture of the N2 (SCTP) interface between the satellite gNodeB (192.168.18.82) and the satellite AMF (192.168.18.80) during autonomous operation. The highlighted packets show the complete NGAP/NAS sequence of a successful IoT UE attach: Registration Request, authentication, NAS Security Mode, Initial Context Setup and PDU Session Resource Setup Response, with no error responses or retransmissions.

The originating IoT UE completed the standard 5G registration and PDU session establishment procedure, authentication, security-context setup and IP allocation from the 10.45.0.0/16 pool, consistently within approximately 0.35-s (Figure 6). The corresponding NGAP/NAS exchange (Figure 7), captured at the N2 interface between the gNodeB and the core, confirms that the procedure follows the expected 3GPP signaling flow without authentication or session-assignment errors. Figure 7 documents this procedure at the packet level: the ordered NGAP/NAS exchange registration request, authentication, NAS security mode, initial context setup, and PDU-session resource setup) completes without error responses or retransmissions. This confirms that the IoT UE performs a standards-compliant 5G registration and session establishment entirely within the satellite segment, i.e., control-plane continuity is preserved during autonomous operation.

### 6.4. Store-And-Forward Data Integrity and Reliability

The S&F mechanism was evaluated by generating IoT data during disconnected operation and persisting it locally within the satellite segment. Data messages were stored using structured payloads that preserved content and associated metadata throughout the disconnected phase.

Upon restoration of backhaul connectivity, the buffered messages were delivered to the destination, enabling end-to-end data delivery without loss or corruption. Experimental validation confirmed that all transmitted messages maintained complete integrity throughout the store-and-forward process. These results demonstrate that integrating S&F functionality at the system level effectively supports delay-tolerant IoT data delivery under intermittent connectivity conditions. S&F store and retrieve operations completed in the order of a few tens of milliseconds (well below 200 ms), i.e., negligible compared with the registration and session-establishment time. Across the 200 simulation runs performed, all buffered messages were retrieved intact, corresponding to a delivery ratio of 100% with no message loss. Across all experimental cycles, stored data was successfully forwarded upon connectivity restoration without any observed loss or corruption, confirming the reliability of the integrated persistence mechanism.

**Figure 8:**
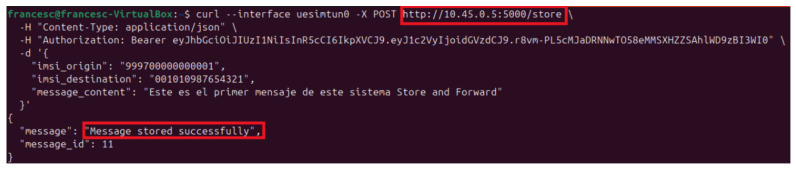
IoT message stored through the S&F REST API.

**Figure 9:**
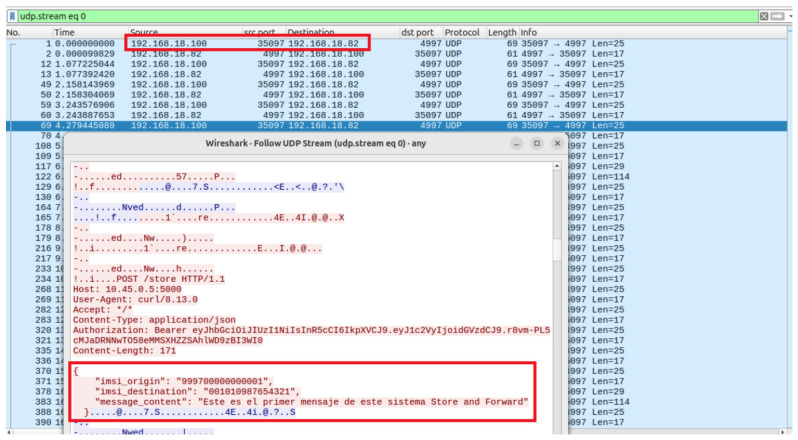
Store-and-Forward persistence during the disconnected phase: an uplink IoT message is buffered locally via the REST API, and the corresponding HTTP/2 transaction is captured at the network level with Wireshark.

Uplink IoT messages generated during the disconnected phase are pushed to the Store-and-Forward service through its REST API after JWT authentication, and are persisted with their content and associated metadata (originating IMSI, timestamp and priority), as shown in Figure 8. Store and retrieval operations exhibited sub-second latency, and the associated HTTP/2 transaction is observable at the network level (Figure 9).

Quantitative indicators are obtained from the recorded experiments without additional runs. The UE registration and session-establishment time is measured from the UERANSIM UE log as the interval between the Initial Registration message and the successful PDU-session setup (Figure 6); the S&F store and retrieve latencies are reported as order-of-magnitude estimates for the local REST operations; and the delivery reliability is reported over the 200 executed runs. Table 8 summarizes these indicators.

### 6.5. Inter-Segment Reintegration and Service Synchronization

The experimental results obtained across disconnected and reconnected operational phases can be abstracted into a conceptual system-level model. Rather than representing isolated experimental observations, the validated behavior reveals two well-defined operational states governing the system dynamics under intermittent connectivity conditions.

**Figure 10:**
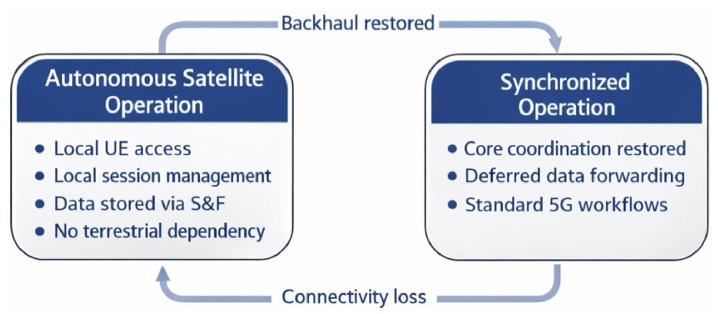
System operational state transition model derived from experimental validation. The system alternates between autonomous and synchronized satellite operation depending on backhaul availability, exhibiting structured, repeatable behavior under intermittent connectivity conditions.

As illustrated in Figure 10, the system alternates between an *autonomous satellite operation* state and a *synchronized operation* state. In the autonomous state, the satellite segment independently supports UE access, session management, and local data persistence via the Store-and-Forward service, without reliance on terrestrial coordination. When backhaul connectivity is restored, the system transitions to the synchronized state, where deferred data forwarding and standard 5G core coordination are reestablished. Conversely, loss of connectivity triggers a transition back to autonomous operation. This state-based interpretation highlights that the proposed architecture exhibits structured and repeatable operational behavior, reinforcing its suitability for real-world NTN-enabled IoT deployments.

A critical aspect of the proposed architecture is the seamless reintegration of distributed network segments upon connectivity restoration. Experimental results show that the satellite segment dynamically reestablishes coordination with the terrestrial core via standard service-discovery mechanisms.

During this synchronized operational phase, stored data is successfully transferred to the terrestrial segment while control-plane coordination resumes without manual intervention. This behavior confirms that the transition between autonomous and synchronized modes can be achieved transparently, preserving service continuity and avoiding disruptions at the UE or application levels.

**Figure 11:**
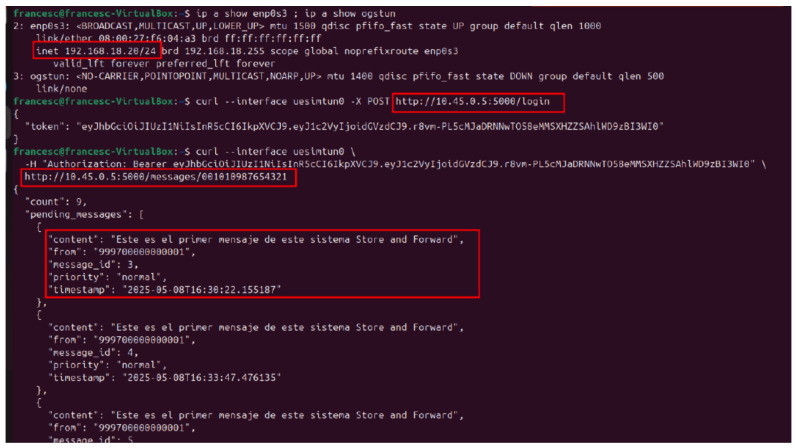
Destination UE retrieves the buffered message after reconnection.

**Figure 12:**
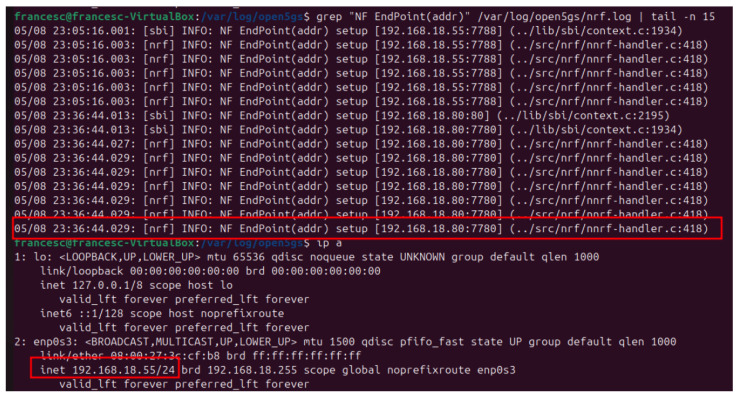
Synchronized operation after backhaul restoration: deferred data is delivered end-to-end to the destination UE, and the satellite AMF is dynamically discovered and registered at the terrestrial NRF through standard service-based mechanisms.

The end-to-end outcome of the synchronized phase is shown in Figure 11: once backhaul connectivity is restored, the satellite AMF is dynamically discovered and registered at the terrestrial NRF through standard service-based mechanisms (Figure 12); the destination UE then retrieves the buffered messages directly from the S&F service, which holds them in a local database on the satellite-side node, through its REST API (Figure 11). The monitoring component described in Section 4.2, intended to autonomously push the messages toward the terrestrial segment on link-up, is part of the implementation but its operation was not separately recorded in these experiments. Across all experimental cycles every stored message was forwarded and delivered without loss or corruption. Table 9 consolidates the validation campaign, mapping each functional criterion to the experimental evidence that supports it; all defined criteria were satisfied, confirming consistent and repeatable behavior across the disconnected and reconnected operational phases.

Connection interruptions are emulated in a controlled and reproducible way by starting and stopping the corresponding services and virtual machines. This is a deliberate methodological choice: it isolates the core-network behavior under validation (registration continuity, data persistence and inter-segment reintegration) from radio and orbital layer variability, so that the observed behavior is attributable to the architecture rather than to link conditions. Within this setup, synchronization recovery is governed by internal, standards-based mechanisms: upon reconnection, the satellite AMF re-registers at the terrestrial NRF through service discovery (Figure 12), and, as reported in Section 6.7, the transient coexistence of two AMF instances requires an SMF context reset before new registrations succeed. External physical factors such as Doppler, visibility-window dynamics, channel impairments, and orbital mechanics act at the radio/link layer: they modulate the frequency and duration of the interruptions, but not the reintegration logic validated here.

### 6.6. System-Level Insights and Limitations

The experimental results highlight several system-level insights. First, distributing selected core functions enables autonomous operation without compromising standard procedures, confirming the practicality of architectural adaptations for NTN scenarios. Second, integrating Store-and-Forward functionality within the core architecture provides an effective mechanism for supporting delay-tolerant IoT services.

However, the results also reveal inherent limitations. The current validation deliberately uses a small number of devices (an originating IoT UE, an auxiliary UE and a destination UE), which is the minimal set needed to exercise the complete end-to-end store-and-forward path; the objective is functional feasibility, not load characterization. At larger device populations, the design does not introduce a new scaling barrier beyond two identifiable points: the S&F persistence and REST layer, currently backed by a lightweight SQLite store, whose throughput bounds the sustainable message rate; and the transient multi-AMF coordination reported in Section 6.7, which currently requires an SMF context reset. Because the architecture reuses the standard 5G service-based architecture, it inherits the horizontal-scaling mechanisms of the 5G core (multiple UPF and SMF instances and NF scaling), and, since the UEs are software-emulated, the ceiling reached in the prototype is the capacity of the emulation host (five virtual machines on a single workstation) rather than a protocol or architectural limit. A dedicated load study with hundreds to thousands of devices is the natural next step to quantify these bounds.

Additionally, the trade-off between autonomy and centralized coordination requires careful functional placement to avoid excessive complexity or synchronization overhead. These aspects represent important directions for future investigation.

### 6.7. Operational Constraints and Deployment Insights

Beyond confirming functional feasibility, the implementation exposed a set of practical constraints directly relevant to the design of distributed 5G core deployments over NTN. First, co-locating a gNodeB with a core instance on a shared network interface leads to SCTP/NGAP port collisions once the default per-function loopback addressing is replaced by a single routable interface; isolating the access node in a separate node removed the conflict and is the recommended deployment pattern. Second, when the satellite AMF is attached to the terrestrial core during the synchronized phase, two AMF instances momentarily coexist and share session context, which blocks new UE registrations; restarting the SMF after the second AMF joins clears the stale context and restores correct operation. These observations indicate that functional placement and multi-AMF coordination are the critical design points when distributing the control plane, consistent with the autonomy/coordination trade-off discussed in Section 3. The system also proved sensitive to the initialization order of the distributed functions. A deterministic startup sequence was required for reproducible end-to-end operation:

1. Start the satellite core (Open5GS network functions).
2. Start the satellite gNodeB.
3. Start the IoT and auxiliary UEs.
4. Exercise the Store-and-Forward store/list operations.
5. Stop the satellite core functions except the AMF and SCP.
6. Start the terrestrial core.
7. Restart the satellite AMF and SCP.
8. Restart the terrestrial SMF.
9. Start the terrestrial gNodeB and the destination UE.
10. Verify connectivity and perform the Store-and-Forward download.

Finally, the scope of the emulation must be kept in mind when interpreting the results: the testbed reproduces the logical connectivity and the full 5G signaling flow, but it does not model the physical radio channel, Doppler effects, or the orbital dynamics of a real LEO platform, and it is not intended to scale to hundreds of nodes. These constraints do not affect the system-level conclusions on autonomy, data persistence and standards-compliant reintegration, but they delimit the boundary between the architectural validation presented here and a future field or hardware-in-the-loop deployment.

Capturing and validating the autonomous, monitor-driven satellite-initiated push end to end (the monitor firing and the push transaction) is left as future work.

## 7. Conclusions and Future Work

This paper has presented a system-level study of a distributed 5G core architecture designed to support delay-tolerant Internet of Things (IoT) services over Low Earth Orbit (LEO) Non-Terrestrial Networks (NTNs). Motivated by the intermittent connectivity and dynamic topology inherent to LEO satellite systems, the proposed architecture distributes selected 5G core network functions across satellite and terrestrial segments and integrates Store-and-Forward (S&F) mechanisms to enable autonomous operation during backhaul disruptions.

Through the implementation of a representative system prototype, the work has experimentally validated the feasibility of autonomous UE registration, session establishment, and reliable data persistence during disconnected operational phases. The results demonstrate that standard 5G procedures can be preserved without protocol extensions, while architectural adaptations at the system level enable resilient operation under intermittent connectivity conditions, in line with the principles outlined in 3GPP Release 19.

From an experimental standpoint, the proposed system exhibits stable and repeatable behavior across all evaluated scenarios. UE registration and session establishment procedures were consistently completed in approximately 0.35 s during autonomous operation, while Store-and-Forward interactions showed sub-second latency for data storage and retrieval. Across all experimental cycles, across the 200 runs performed, no data loss or corruption was observed (a 100% delivery ratio), confirming the reliability of the integrated persistence mechanisms under intermittent backhaul conditions.

Beyond functional validation, this study provides system-level insights into the design trade-offs associated with distributed 5G core deployment, particularly regarding functional placement, autonomy, and centralized coordination. Future work will address scalability for multi-satellite and multi-gateway scenarios, investigate intelligent, traffic-aware Store-and-Forward strategies, and explore dynamic orchestration mechanisms, as well as security and resilience aspects tailored to spaceborne deployments. Regarding security, future work may explore mechanisms to protect the inter-segment synchronization and data transfer. While quantum key distribution has been studied in this context and its vulnerabilities analyzed [17], such schemes are likely disproportionate to the threat model and resource constraints of low-rate, delay-tolerant IoT over resource-limited satellites; a lightweight, standards-based protection of the service-based interfaces is therefore a more proportionate near-term direction.

## Figures and Tables

**Table 1:** Hardware and software configuration of the experimental testbed.

Component	Specification	Version
Host processor	Intel Core i7-9700 (8 cores, 8 threads, up to 4.7 GHz)	—
Host memory	32 GB DDR4	—
Host storage	1 TB SSD	—
Host operating system	Ubuntu	20.04.6 LTS
Hypervisor	Oracle VirtualBox (nested virtualization enabled)	7.1.4
Virtual machines	5 × Ubuntu; 3 vCPU, 4 GB RAM, 20 GB disk each	24.04.1 LTS
Container runtime	Docker	24.0.5
5G core	Open5GS (service-based architecture)	v2.7.2
RAN/UE emulation	UERANSIM (gNodeB and UE)	v3.2.6
Subscriber database	MongoDB	6.0
Traffic analysis	Wireshark (NGAP, NAS, GTP, SCTP, HTTP/2)	4.2.5
S&F service runtime	Python	3.12
S&F REST API	Flask	3.0.3
S&F persistence	SQLite	3.45.1
REST test client	curl	8.5.0
Management network	Internal VirtualBox network 192.168.18.0/24	—
PDU session subnet	10.45.0.0/16 (dynamically assigned to UEs)	—

**Table 2:** Virtual machine roles and management-network IP allocation.

VM	Role and Hosted Services	IP Address
VM1	Satellite core-Open5GS network functions	192.168.18.80
VM2	Satellite gNodeB + S&F service (Docker) + auxiliary UE	192.168.18.82
VM3	Originating IoT UE (UERANSIM)	192.168.18.100
VM4	Terrestrial core-Open5GS network functions	192.168.18.55
VM5	Terrestrial gNodeB + destination UE (UERANSIM)	192.168.18.20

**Table 3:** Service-Based Interface (SBI) configuration of the Open5GS network functions in the satellite and terrestrial segments. The UPF does not expose an SBI: SMF–UPF signalling uses PFCP over UDP (port 8805).

	Satellite Core		Terrestrial Core	
NF	IP	SBI Port	IP	SBI Port
AMF	192.168.18.80	7780	192.168.18.55	7780
AUSF	192.168.18.80	7781	192.168.18.55	7781
BSF	192.168.18.80	7782	192.168.18.55	7782
NRF	192.168.18.80	7777	192.168.18.55	7777
NSSF	192.168.18.80	7784	192.168.18.55	7784
PCF	192.168.18.80	7785	192.168.18.55	7785
SCP	192.168.18.80	7799	192.168.18.55	7799
SMF	192.168.18.80	7788	192.168.18.55	7788
UDM	192.168.18.80	7789	192.168.18.55	7789
UDR	192.168.18.80	7790	192.168.18.55	7790
UPF	192.168.18.80	—	192.168.18.55	—

**Table 4:** Provisioned subscriber identities. All UEs share the same authentication material (key and OPc) and the null SUCI protection scheme to simplify the controlled experiment.

Role	IMSI	Segment
Originating IoT UE	999700000000001	Satellite
Auxiliary UE (S&F relay)	999700000000002	Satellite
Destination UE	999700000000003	Terrestrial

**Table 5:** Assignment of roles, management-network addresses and PDU-session addresses across the distributed testbed.

Element	Role	Network IP	Session IP
IoT UE (satellite)	Originating device	192.168.18.100	10.45.0.2
Auxiliary UE (satellite)	S&F reception/forwarding	192.168.18.82	10.45.0.5
Satellite gNodeB	Satellite access node	192.168.18.82	—
Satellite AMF	Satellite core	192.168.18.80	—
Satellite SMF	Satellite core	—	10.45.0.1
Terrestrial gNodeB	Terrestrial access node	192.168.18.20	—
Destination UE	Destination device	192.168.18.20	10.45.0.6
Terrestrial AMF	Terrestrial core	192.168.18.55	—
Terrestrial SMF	Terrestrial core	—	10.45.0.1
Terrestrial UPF	User plane	—	—

**Table 6:** REST endpoints exposed by the Store-and-Forward service. The service is containerized (Docker), persists messages in a lightweight SQLite database, and secures requests with JWT authentication.

Endpoint	Method	Description
/store	POST	Store an uplink IoT message during a backhaul outage
/messages/<imsi>	GET	Retrieve pending messages for a given subscriber
/ack/<message_id>	POST	Acknowledge reception of a forwarded message
/health	GET	Report service liveness/status

**Table 7:** Experimental Configuration Summary.

Aspect	Configuration
Deployment approach	Virtualized distributed system
Network segments	Satellite segment and terrestrial segment
Satellite segment role	Autonomous access, session control, S&F
Terrestrial segment role	Centralized core and data delivery
Number of logical nodes	Multiple distributed network entities
Operational modes	Disconnected (autonomous) and connected phases
Data persistence	Local storage during backhaul outages
UE roles	Originating, intermediary, and destination devices

**Table 8:** Timing indicators and delivery reliability. Latency values are measured from the representative logged run (Figure 6); delivery reliability is reported over the 200 executed runs.

Metric	Value	Basis
UE registration procedure	≈70 ms	Figure 6 (representative)
PDU-session establishment	≈280 ms	Figure 6 (representative)
Registration + session (total)	≈0.35 s	Figure 6 (representative)
S&F store operation (POST)	tens of ms (<200 ms)	REST *
S&F retrieve operation (GET)	tens of ms (<200 ms)	REST *
Delivery ratio	100% (200/200, 0 loss)	200 runs

* Order-of-magnitude estimate for local REST operations.

**Table 9:** Validation criteria and experimental outcomes. Each criterion is supported by logs, terminal captures and/or Wireshark traces obtained from the prototype.

Validation Criterion	Experimental Evidence	Met
Satellite core operation and registration of elements (gNodeB, IoT UE, auxiliary UE)	Figure 4, Figure 5 and Figure 6	Yes
Local buffering and forwarding of IoT data with S&F	Figure 8 and Figure 9	Yes
Terrestrial core operation and registration of elements	Terrestrial core NF status and gNodeB/UE registration logs	Yes
Delivery of buffered messages to the destination UE	Figure 11	Yes
Distributed architecture and dynamic registration of the satellite AMF	Figure 12	Yes

## Data Availability

The original contributions presented in this study are included in the article. Further inquiries can be directed to the corresponding author. The data are not publicly available due to privacy.

## Abbreviations

The following abbreviations are used in this manuscript:

3GPP	3rd Generation Partnership Project
5G	Fifth Generation
AMF	Access and Mobility Management Function
CP	Control Plane
DTN	Delay-Tolerant Networking
EPC	Evolved Packet Core
GSO	Geostationary Orbit
IoT	Internet of Things
ISL	Inter-Satellite Link
LEO	Low Earth Orbit
LoRa	Long Range
LoRaWAN	Long Range Wide Area Network
LPWAN	Low-Power Wide-Area Network
MME	Mobility Management Entity
NB-IoT	Narrowband Internet of Things
NR	New Radio
NTN	Non-Terrestrial Network
PDN	Packet Data Network
PDU	Protocol Data Unit
RAN	Radio Access Network
RRM	Radio Resource Management
S&F	Store-and-Forward
SMF	Session Management Function
TAU	Tracking Area Update
TR	Technical Report
UE	User Equipment
UPF	User Plane Function

## References

[1] 3GPP (2021). Solutions for NR to Support Non-Terrestrial Networks (NTN); Technical Report TR 38.821, 3rd Generation Partnership Project; Version 16.1.0, Release 16. https://www.3gpp.org/DynaReport/38821.htm.

[2] Hanifah S., Kurnianingrum I.P., Jamil A.R.A., Yovita L.V., Wibowo T.A. Performance Evaluation of Real Delay Tolerant Network in Rural Environments. Proceedings of the 2025 IEEE International Symposium on Future Telecommunication Technologies (SOFTT).

[3] Zarri M., Kim D. (2025). How Satellites Enable Mobile Network Evolution: 3GPP Release 19 Progress of Satellite Integration. IEEE Commun. Stand. Mag..

[4] 3GPP (2024). Study on Integration of Satellite Components in the 5G Architecture; Phase 3. Technical Report TR 23.700-29, 3rd Generation Partnership Project (3GPP), Sophia Antipolis, France. https://www.3gpp.org/DynaReport/23700-29.htm.

[5] Carneros P.G., Baeza V.M. (2026). Probabilistic Link Opportunity Prediction for GNSS-Free NB-IoT Ground Devices in LEO Satellite Networks. IEEE Commun. Lett..

[6] Yoo H., Park M., Ko N. A Mobile System Architecture for Store-and-Forward Operation in Non-Terrestrial Networks. Proceedings of the 2025 Sixteenth International Conference on Ubiquitous and Future Networks (ICUFN).

[7] Choi J. Mitigating Latency for LEO Satellite Internet of Things via Inter-Satellite Links. Proceedings of the 2025 IEEE 36th International Symposium on Personal, Indoor and Mobile Radio Communications (PIMRC).

[8] Andi T., Adi P.D., Hirsi A., Hermida I.D., Andayani D.G., Siregar V.M., Sadiyah L., Lestari P. Performance Evaluation of Store-and-Forward Method for LoRaWAN Satellite Communication. Proceedings of the 2025 International Conference on Information Technology and Computing (ICITCOM).

[9] Zong J., Nie H., Qi W., Wang H., Xia X. Architecture and Procedure Enhancements for Store and Forward Satellite Operations. Proceedings of the 2025 International Wireless Communications and Mobile Computing (IWCMC).

[10] Lu Y., Zhao Y., Sun F., Qin D. (2016). Complexity of Routing in Store-and-Forward LEO Satellite Networks. IEEE Commun. Lett..

[11] Rahim S.N.M., Johari J., Rahim S.A.E.A., Jusoh M.H. (2018). Estimation of Communication Link on Ground Sensor Terminal (GST) System for Nanosatellite (UiTMSAT-1) Store-and-Forward Mission. Proceedings of the 2018 IEEE 8th International Conference on System Engineering and Technology (ICSET), Bandung, Indonesia, 15 October 2018.

[12] Kellermann T., Pueyo Centelles R., Camps-Mur D., Ferrús R., Guadalupi M., Calveras Augé A. (2022). Novel Architecture for Cellular IoT in Future Non-Terrestrial Networks: Store and Forward Adaptations for Enabling Discontinuous Feeder Link Operation. IEEE Access.

[13] Monzon Baeza V., Rigazzi G., Aguilar Romero S., Ferrus R., Ferrer J., Mhatre S., Guadalupi M. IoT-NTN Communications via Store-and-Forward Core Network in Multi-LEO-Satellite Deployments. Proceedings of the 2024 IEEE 35th International Symposium on Personal, Indoor and Mobile Radio Communications (PIMRC).

[14] Saglam M.İ. An Enhanced Paging Method for Store-and-Forward Internet of Things Non-Terrestrial Networks. Proceedings of the 2025 12th International Conference on Electrical and Electronics Engineering (ICEEE).

[15] Open5GS Project (2024). Open5GS: Open Source 5G Core Network. https://open5gs.org.

[16] UERANSIM Project (2024). UERANSIM: Open Source 5G UE and gNodeB Simulator. https://github.com/aligungr/UERANSIM.

[17] Pljonkin A., Petrov D., Sabantina L., Dakhkilgova K. (2021). Nonclassical Attack on a Quantum Key Distribution System. Entropy.

